# Environmental and Occupational Health Response to SARS, Taiwan, 2003

**DOI:** 10.3201/eid1007.030728

**Published:** 2004-07

**Authors:** Eric J. Esswein, Max Kiefer, Ken Wallingford, Greg Burr, Lukas Jyhun-Hsiarn Lee, Jung-Der Wang, Shun Chih Wang, Ih-Jen Su

**Affiliations:** * National Institute for Occupational Safety and Health (NIOSH), Denver, Colorado, USA;; † NIOSH, Atlanta Field Office, Atlanta, Georgia, USA;; ‡ NIOSH, Cincinnati, Ohio, USA;; § Department of Health, Taipei, Taiwan;; ¶ National Taiwan University, Taipei, Taiwan;; # Institute of Occupational Safety and Health, Taipei, Taiwan;; ** Taiwan Center for Disease Control, Taipei, Taiwan

**Keywords:** severe acute respiratory syndrome, SARS, Taiwan, hospitals, industrial hygiene, healthcare facilities, ventilation, personal protective equipment, infection control, airborne infection isolation rooms, perspective

## Abstract

Environmental and Occupational Health Industrial hygiene emergency response to SARS in Taiwan.

Industrial hygiene specialists from the National Institute for Occupational Safety and Health (NIOSH) visited hospitals and medical centers throughout Taiwan. They assisted with designing and evaluating ventilation modifications for infection control, developed guidelines for converting hospital rooms into SARS patient isolation rooms, prepared designs for the rapid conversion of a vacated military facility into a SARS screening and observation facility, assessed environmental aspects of dedicated SARS hospitals, and worked in concert with the Taiwanese to develop hospital ventilation guidelines. We describe the environmental findings and observations from this response, including the rapid reconfiguration of medical facilities during a national health emergency, and discuss environmental challenges should SARS or a SARS-like virus emerge again.

The outbreak of severe acute respiratory syndrome (SARS) placed unprecedented demands on healthcare practitioners, healthcare institutions, and public works personnel worldwide. Taiwan reported the third largest number of SARS infections and deaths, followed by Hong Kong and mainland China (*1*). At the request of the Taiwan Department of Health, the Centers for Disease Control and Prevention (CDC) sent staff to Taiwan that included epidemiologists, infectious disease experts, and environmental and logistical specialists. Industrial hygienists were requested to investigate and help develop guidance for hospitals about patient isolation rooms, personal protective equipment, general infection control, and hospital health and safety.

From April 29, 2003, through June 13, 2003, four industrial hygienists from the National Institute for Occupational Safety and Health (NIOSH) conducted environmental assessments of 32 hospitals and medical centers throughout Taiwan. NIOSH staff were deployed serially (a team of two initially and later, two persons at two different times) within larger teams stationed first in Taipei and then in Kaohsiung. CDC personnel worked together with Taiwanese scientists from the Taiwan Department of Health (DOH), Taiwan Center for Disease Control, National Taiwan University, College of Public Health (NTUCPH), and the Taiwan Institute of Occupational Safety and Health (Taiwan IOSH). During the peak of the SARS epidemic, CDC environmental support focused on immediate steps to isolate SARS patients, protect healthcare workers and other personnel during fever screening and patient care, and provide advice on disinfection, direct contact, and airborne precautions. As the epidemic waned, efforts turned to assessing the implementation of infection control practices, strategies for handling future SARS patients, facility designs for effective patient isolation and fever screening stations, personal protective equipment practices, and training of healthcare workers. Thirty-two hospitals or medical centers that were either accepting and treating SARS patients or were under consideration for use as dedicated SARS treatment facilities in anticipation of a more widely disseminated epidemic were visited.

We describe an uncharacteristic industrial hygiene and public health response that occurred under conditions of a national health emergency. The circumstances, an evolving epidemic occurring in a worldwide atmosphere of tremendous uncertainty, elicited a unique response, which went beyond the traditional industrial hygiene investigative model. The requirement that the response teams deploy to the field on short notice, swiftly conduct multiple site investigations, and provide expedient recommendations on multiple topics in various healthcare environments, increased the challenges of this assignment. Although environmental findings and observations from hospital site visits and the rapid reconfiguration of medical facilities into dedicated SARS patient facilities are described, they are not necessarily prescriptive. Global health emergencies demand quick action, which was the case for the SARS epidemic. We describe the environmental challenges that could occur should SARS or a SARS-like virus emerge again. Prescriptive environmental guidelines are available elsewhere (*2*). Comprehensive ventilation engineering or facility evaluations of every hospital or healthcare facility were not possible to obtain.

## Chronology and Methods

Two staff members arrived April 29, 2003, and met with CDC team members to coordinate response roles. Meetings were arranged with Taiwanese government officials and scientists, including Taiwan DOH, Taiwan Centers for Disease Control, NTUCPH, and Taiwan IOSH. The need was agreed on for controls to isolate SARS patients and ensure protection of healthcare workers and other personnel exposed to patients suspected or known to be infected with SARS. A four-phase environmental approach was developed, which included the following: 1) conduct environmental needs assessments for healthcare facilities; 2) conduct environmental field assessments for healthcare facilities; 3) develop written environmental guidelines for healthcare facilities; and 4) conduct environmental audits of selected healthcare facilities.

## Environmental Needs Assessment for Healthcare Facilities

In collaboration with Taiwanese scientists, a needs assessment for healthcare facilities was conducted to understand the most important environmental issues for controlling the spread of SARS in hospitals. Additional airborne infection isolation rooms (AIIRs) were needed on a temporary and a permanent basis. Developing environmental guidelines for design and evaluation of such rooms was a priority. Training healthcare workers in appropriate use of personal protective equipment and infection control practices was also an immediate need at the hospital level.

## Environmental Field Assessments of Healthcare Facilities

Field assessments of healthcare facilities were conducted to better understand standard design and ventilation parameters and how existing AIIRS were configured. The Taiwan Center for Disease Control selected the facilities to represent a cross-section of national, municipal, military, and private hospitals. Environmental field teams typically included NIOSH specialists, a Taiwan Center for Disease Control physician, an occupational health specialist, a Taiwan IOSH engineer, and a doctoral student from NTUCPH. Discussions were held with senior hospital management to understand general hospital configurations, total patient and AIIR capacity, infection control practices, and to determine if SARS patients were being treated at the facility. Ventilation designs were discussed with facilities staff, ventilation drawings were reviewed, and AIIRS, if present, were observed. Discussions with staff during the site visit allowed the team to evaluate environmental issues regarding the expedient addition of isolation rooms, issues affecting infection control, and the selection and use of appropriate personal protective equipment. Table 1 describes the first 10 hospitals and 2 fever clinics that were visited from April 29, 2003, through May 13, 2003, in and around Taipei and Kaoshiung, Taiwan.

**Table 1:** Environmental findings from hospitals and medical centers in Taipei, Taiwan^a,b^

Hospitals	IRs	Single pass AHU	HEPA	UV	Pressure monitors	BP review	Comments
T-1	10^c^ 27^d^	N	Y	Y	Y	Y	Medical center (largest healthcare facility category in Taiwan). Affiliated with Taiwan University School of Public Health. Two visits made by CDC team.
T-2	9^c^ 3^d^	Y	Y	Y	Y	Y	Two visits made by CDC team. Three IRs were constructed within 1 week for the ER.
T-3	0	Y	Y	N	N	Y	No IRs. Instead windows in SARS patient rooms kept open. Suggested closing windows and adjusting thermostat and fan settings in patient rooms to increase negative pressure.
T-4	12^c^ 12^c^	Y	Y	N	Y	Y	Suburban hospital, scheduled to receive SARS patients.
T-5	108–120^d^	Y	Y	Y	Y	Y	Under conversion to a designated SARS hospital.
T-6	1^c^ 6^d^	Y	Y	Y	Y	Y	Rural hospital approximately 2 hours from Taipei City.
T-7	10^d^	Y	Y	Y	Y	Y	Suburban hospital where non-SARS patients from Ho-Ping Hospital (facility closed during the SARS outbreak) would be transferred.
T-8	56^d^	Y	Y	Y	Y	Y	Medical center with entire building being converted to a SARS wing.
T-9	77^d^	Y	Y	Y	Y	Y	Formerly closed military hospital, this facility was under conversion to designated SARS hospital.
T-10	77^d^	Y	Y	Y	Y	Y	Medical center and only private hospital of the group visited. The newly installed single-pass ventilation system with HEPA/UV filtration was excellent.
Fever clinics	^e^	Y	^e^	^e^	Y	N	Under construction in paved parking areas adjacent to the hospital.

^a^All hospitals listed strongly suggested (or required) wearing filtering face-piece respirators when entering the hospital. Persons entering these facilities were screened for fever before entering the facility (using infrared skin or tympanic membrane sensors) and dispensed sanitizing gels or disposable hand-cleaning wipes. ^b^IRs, isolation rooms; AHU, air handling units; BP, HEPA, high efficiency particle aerosol; UV, ultraviolet germicidal irradiation lamps; blue print or engineering designs plans available for review; Y, yes; N, no; CDC, Centers for Disease Control and Prevention; SARS, severe acute respiratory syndrome. ^c^IRs available during initial visit (numbers include IRs in all areas of the hospital). ^d^IRs planned for completion (within weeks). ^e^IRs planned ranged from 2 to 6 per location. Ventilation in IRs ranged from simple (standard bathroom exhaust fans, without HEPA or UV treatment of exhaust air) to well-designed single-pass exhaust air systems with HEPA/UV treatment of exhaust air. No IRs present at time of visit. Hospital used standard patient rooms for SARS patients, providing 100% supply air, with exterior windows remaining open. Pressure differentials between patient rooms and remainder of floor where minimal hospital evaluation not possible. Determination made on the number or adequacy of IRs available. The NIOSH/CDC team recommended that this hospital not be used for SARS patients based on lack of information regarding ventilation system. Fever clinics included tented areas or small buildings (generally under construction) outside hospital ERs used to screen for fever and other symptoms to identify possible SARS-infected patients before entering the hospital.

## Environmental Guidelines for Healthcare Facilities

A Taiwan IOSH engineer, 27 Taiwanese public health and ventilation specialists, and a NIOSH industrial hygiene specialists developed Guidelines for the Integrity and Inspection of SARS Isolation Wards. The information was to be used to construct permanent infrastructure and included the appropriate layout of a SARS treatment room and ward, ventilation design for sufficient negative pressure in AIIRs, and filtration and treatment of exhausted air. This document also included measures to enhance infection control and protect healthcare workers during maintenance of the isolation room and ventilation system. The document was written in Chinese and was posted on Web sites of the Taiwan Center for Disease Control and IOSH and distributed to healthcare facilities (http://www.iosh.gov.tw/eversion/sarse.htm). The Taiwan Center for Disease Control also used this document in training sessions for healthcare managers and workers throughout Taiwan.

Because of an immediate need for simple ventilation modifications to reconfigure existing patient rooms to serve as AIIRs, NIOSH staff developed a list of possible modifications to create and confirm negative pressure in typical patient rooms. Applications varied by facility and included increasing exhaust air volume with the addition of assist fans, ways to reduce room leakage and confirm negative pressure, such as using flutter strips on doors or pressure gauges on walls. More complex modifications that used self-contained air exchange, HEPA filtration, and ventilation units were also included.

## Environmental Audits of Selected Healthcare Facilities

Audits evaluated how well the SARS isolation ward guidelines had been implemented. Hospital managers were contacted, and walkthrough surveys were arranged for investigators. The general objectives were to determine the adequacy of AIIRs, provide guidance for construction or conversion of existing patient rooms into AIIRs, provide technical assistance on ventilation and other controls (e.g., isolation, procedures, and training), evaluate appropriate use of personal protective equipment, and provide guidance on various environmental considerations for infection control.

## Kaohsiung County, Tainan, and Chai-Yi

Twelve hospitals and medical centers in southern Taiwan were visited May 17–25, 2003. Meetings were held with facilities and ventilation engineers, hospital administrators, and medical and nursing staff to understand the number, type, and location of AIIRs. Mechanical drawings, blueprints, and ventilation testing reports were reviewed, and walkthrough surveys were conducted. Visible smoke testing was performed to evaluate pressurization between nurses' stations and SARS patients' wards and for as many individual patient rooms as possible. When possible, air handling units, HEPA filters, outside air intakes, and rooftop exhaust ductwork were inspected. Infection control procedures and personal protective equipment use were reviewed, including availability, staff knowledge regarding proper use, and implementation.

Standard versus simple negative-pressure isolation rooms were observed. Typically, standard isolation rooms had an anteroom for use by healthcare workers to put on and take off personal protective equipment, a digital or analog pressure manometer mounted outside the door, hard rather than suspended ceilings, and walls that extended floor-to-ceiling. Headwall and utility penetrations were sealed to reduce leakage, maintain negative pressure, and control airflow. Constant volume air-handling units were commonly used and configured to operate in the single pass mode. Most air-handling units could be tested and balanced to maintain negative pressure of 2.5 to 20 pascal (0.008–0.08 inches of water). Exhaust for standard AIIRs were often configured with HEPA filtration. Some facilities installed ultraviolet germicidal irradiation (UVGI) lamps in HEPA filter banks, although the efficacy of using UVGI for control of SARS-associated coronavirus was uncertain.

Most of the simple isolation rooms had been designed for isolating patients with infectious diseases such as scabies. These rooms lacked an anteroom, generally had window-mounted air-conditioning units, and small (1,000–2,000 ft^3^/min) window-mounted vane axial fans for negative pressure. Wall penetrations were typically not sealed, manometers were generally not present, and ceilings were not hard. Unfiltered room air was exhausted outdoors through windows.

Aggressive infection control measures were evident in all hospitals. Hand sanitation stations with automated dispensers were abundant, especially on nurses' wards, at elevator landings, and at every hospital entrance. Infection control staff dispensed sanitizing gels and cloth or disposable hand-cleaning wipes. Infection control personnel were also stationed at hospital entrances and screened for fever by measuring forehead skin temperatures. One hospital installed forward-looking infrared scanning cameras that displayed temperatures on TV monitors next to nurses' stations. Visitors or staff with fever were denied entrance and sent to fever clinics outside the hospital for medical follow-up. Healthcare workers, hospital staff, and visitors all wore filtering face-piece surgical masks or respirators of varying brands and efficiencies (N95 to N100). Most hospitals cordoned a gurney pathway from ambulance entrances to an elevator landing, where a designated and preprogrammed elevator transported SARS patients to SARS wards. When meetings were conducted, all participants wore respirators or masks, and handshaking was minimized or discouraged.

Appropriate protective equipment (including eye protection, protective suits, aprons, gloves, head and foot coverings, and respirators) was widely available and healthcare workers were knowledgeable about their use. In many nurses' stations, posters describing standard operating procedures for SARS protective equipment were present. Environmental findings were discussed at a closing meeting, and written reports were later sent to each hospital. Table 2 summarizes general environmental aspects of 10 hospitals investigated in southern Taiwan and provides examples of recommendations provided to these hospitals.

**Table 2:** Environmental findings from southern Taiwan hospitals and medical centers in Kaohsiung, Tainan, and Chia-Yi^a^

Hospital	IRs	Single pass AHU	HEPA	UV	Pressure monitors^b^	BP review	Negative pressure in IRs?	Recommendations and notes
A	10 (S)	Y	N	N	N	Y	Y	^c^
B	6 (s)	N	N	N	N	Y	N	^d^
C	4 (S) 2 (s)	Y	Y	N	Y	Y	Y	^e^
D	3 (S) 9 (s)	Y	Y	N	Y	Y	Y	
E	29 (s)	Y	N	N	Y	Y	N	^f^
F	2 (S) 72 (s)^g^	N	N	N	N	Y	N	
G	20	Y	N	N	Y	Y	Y	^h^
H	20	Y	N	N	Y	Y	Y	^i^
I	2 (S) 20 (s)	Y	N	N	Y			^j^
J	8 (S) 19± (S)	Y	N	N	Y	Y	Y	^k^

^a^IR, isolation rooms; AHU, air handling units; UV, ultraviolet germicidal irradiation; BP, blueprint or engineering designs plans available for review; S, standard isolation room; s, simple isolation room; Y, yes; N, no. ^b^Pressure monitors were either digital or analog manometers installed outside patient room. ^c^Repair collapsed rooftop exhaust stack. ^d^Inspect isolation rooms for leakage; four rooms not negatively pressurized. ^e^Ensure outdoor air intakes are not in proximity to exhaust fans for simple isolation rooms. ^f^32% isolation rooms were not negatively pressurized, no ICU, not recommended for SARS patients. ^g^Proposed for construction as of 5/2003. ^h^Ventilation system needs balancing/modification (negative pressure varied from –1.4 to –22 pascal). Extend rooftop exhaust stacks, establish standard operating procedures for personal protective equipment use, require handwashing for all hospital contractors. ^i^Modify 2-way switches in simple isolation rooms so that fans cannot operate in reverse, replace wooden doorknobs with metal on SARS patient ward. ^j^Modify 2-way switches in simple isolation rooms so that fans cannot operate in reverse, position patient beds with head of bed near source of room exhaust for increased isolation, seal wall, window, and ceiling penetrations in simple isolation rooms. ^k^Modify 2-way switches in simple isolation rooms so that fans cannot operate in reverse, seal windows in simple isolation rooms to enhance negative pressure.

## Kaohsiung SARS Screening and Observation Facilities

Two proposals for the construction of specialized SARS screening and isolation facilities in southern Taiwan were reviewed by NIOSH and Taiwanese environmental specialists. One proposal considered configuring arrays of shipping containers (widely available in this port city) into patient AIIRs linked in hub-and-spoke fashion by a central nurses' station. This proposal was not recommended because of uncontrolled solar loads on the containers and feasibility issues for patient emergency medical treatment procedures. The second proposal was to convert vacated military barracks into a SARS patient–screening facility. An ambitious timeline required converting an open bay barracks into 20 individual patient isolation rooms and ultimately converting another barracks of similar design for a total of 40 beds. Demolition and reconstruction were to be completed and a functional facility available within 48 hours. A meeting was held May 22, 2003, to tour the site, sketch a preliminary design, and provide verbal recommendations for a proposed redesign of the open bays into an 18-bed facility, configured into simple patient isolation rooms. Demolition began immediately. The responders provided a detailed guidance document the following day, which outlined the following areas: facility design, construction, and renovation specifications for patient rooms; ventilation specifications, including hood designs to improve axial exhaust fans; fire, safety, and environmental guidelines; infection control practices for patients and healthcare workers; placement of sanitary facilities; and patient and staff traffic flow through the facility.

**Figure 1:**
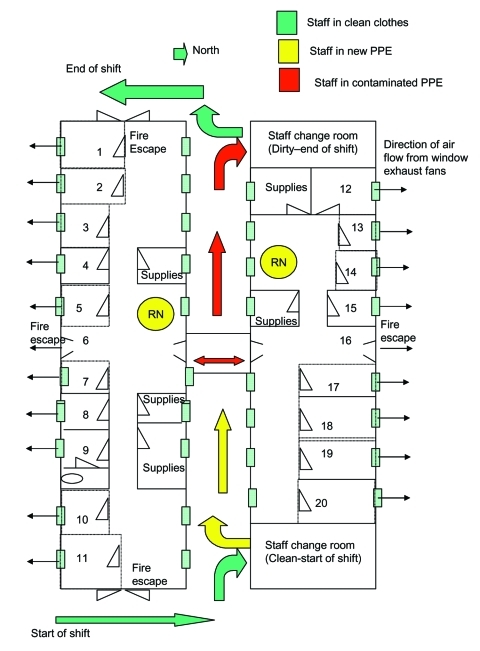
Kaoshsiung SARS fever screening and observation facility, design layout and staff flow diagram. PPE, personal protective equipment. From: Recommendations for Design of a SARS Patient Screening, Isolation and Care Facility. Bloland PB, Esswein EJ, and Wong W; 5/23/2003.

This facility was dedicated on May 28, 2003, substantially increasing the number of AIIRs available in southern Taiwan. The facility could also function as a quarantine station if needed. The floor plan of this converted barracks building is shown in Figure 1.

## Kaohsiung County, Taidong, Taichung, Hualien, and Taipei

The Taiwan DOH issued a directive on April 23, 2003, mandating the establishment of 11 dedicated SARS hospitals and medical centers geographically dispersed throughout Taiwan (Table 3). General care hospitals were directed to treat mild SARS patients (those not on ventilators and with substantial pulmonary capacity) and patients undergoing rehabilitation; patients who needed ventilation were sent to medical centers with intensive care units. SARS-designated hospitals ceased providing general patient care and began complying with the AIIR construction schedule, including reengineering or installation of new ventilation equipment. After the design phase, hospitals completed the conversion (start of construction to patient acceptance) on an average of 13 days, resulting in a total of 698 negative-pressure AIIRs constructed by the completion of the nationwide project.

**Table 3:** Designated SARS Hospitals, Taiwan^a^

Hospital	Hospital type	Location	Start 2003	Completion 2003	Days	No. isolation rooms
DSH-1	Referral	Northern Taiwan	5/8	5/19	11	102 Patient rooms 9 ICU beds 1 Operation room 1 Dialysis room
DSH-2	General care	Northern Taiwan	5/7	5/20	13	92
DSH-3	General care	Northern Taiwan	5/28	6/30	32	77 Patient rooms (119 beds)
DSH-4	Referral	Central Taiwan	5/21	6/6	15	40 Patient rooms 6 ICU beds
DSH-5	General care	Central Taiwan	5/24	6/7	13	42
DSH-6	General care	Southern Taiwan	5/24	6/3	9	53
DSH-7	General care	Southern Taiwan	5/23	6/1	8	83
DSH-8	Referral	Southern Taiwan	5/22	6/4	12	72 Patient rooms 6 ICU beds 1 Operation room 1 Dialysis room
DSH-9	General care	Southern Taiwan	5/23	6/6	13	77
DSH-10	General care	Eastern Taiwan	5/27	6/1	15	28
DSH-11	General care	Eastern Taiwan	5/27	6/1	4	32

^a^SARS, severe acute respiratory syndrome; DSH, dedicated SARS hospital; ICU, intensive care unit.

**Figure 2:**
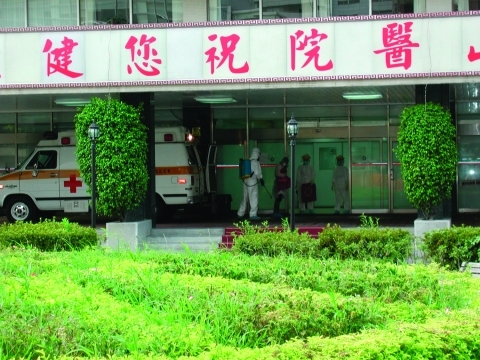
Hospital worker in full personal protective equipment disinfects ambulance and hospital at Song Shan Hospital after delivery of a suspected severe acute respiratory syndrome patient, June 2003. Photograph courtesy of Max Kiefer, Centers for Disease Control and Prevention, Atlanta, GA.

From May 31 through June 10, 2003, a total of 11 dedicated SARS hospitals were evaluated to assess AIIRs and wards, infection control practices, healthcare worker and patient entrance and egress pathways, protective equipment practices, and healthcare worker training (Figure 2). As part of the arrangement by the Taiwan Center for Disease Control for the hospital site visits, hospital management were given a questionnaire that requested design criteria, number of isolation rooms, personal protective equipment requirements, and ventilation specifications. Evaluations began with an opening conference with administrative, engineering, infection control, and healthcare worker staff. Hospital objectives and the status of the hospital modifications were discussed, blueprints were reviewed, ventilation system and AIIR design were discussed, healthcare worker training was reviewed, and the personal protective equipment protocol was observed. A walkthrough of the patient and healthcare worker pathway was conducted, including an inspection of the various isolation gradients (nurses' stations, change rooms, isolation ward corridor, anterooms, and AIIRs). Visible smoke was used to evaluate the pressurization of AIIRs and to assess airflow patterns both within the IR and the ventilation zones and the ventilation system (supply air location, exhaust discharge, HEPA filters, and UVGI) was inspected. Maintenance practices, establishment of an infection control department, location of hand-washing stations, room pressure monitors, and availability of personal protective equipment were reviewed. Closing meetings were held with hospital management and healthcare worker staff to review findings and recommendations and followed up with written reports.

The Taiwan Industrial Technology Research Institute, Taiwan IOSH, and Taiwan Center for Disease Control provided general facility design specifications for these hospitals and other general care facilities treating SARS patients. Most hospitals contracted with architectural and engineering firms to manage design and reconstruction. A national hospital steering committee was formed to guide this effort. Design criteria included specifications from existing CDC tuberculosis guidelines (*3*), the American Society of Heating, Refrigerating and Air-Conditioning Engineers Design Manual for Hospitals and Clinics (*4*), and the American Institute of Architects Guidelines for Hospitals and Health Care Facilities (*5*).

**Figure 3:**
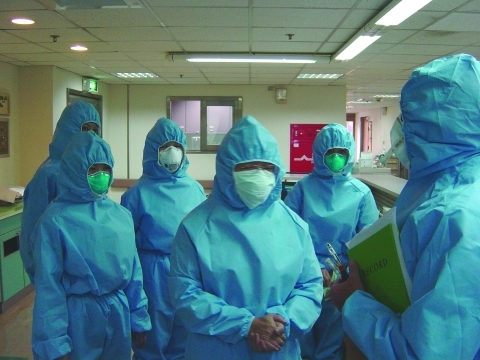
Hospital workers in Kaohsiung, Taiwan, listen to a summary of findings from walkthrough survey and pressurization testing on a severe acute respiratory syndrome patient ward.

**Figure 4:**
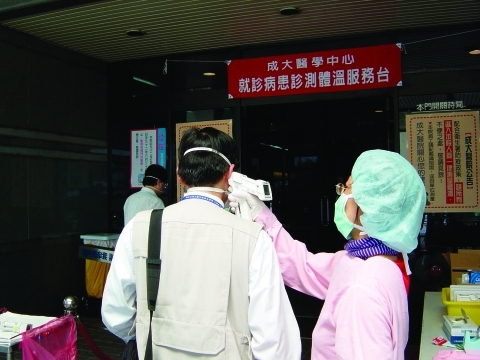
A Center for Disease Control Taiwan investigator is screened for fever before entering a healthcare facility in Kaohsiung.

Massive infrastructure modifications were necessary to achieve the desired objectives of increased capacity to triage and treat seriously ill patients and at the same time protect healthcare workers. Examples include the reconfiguration of the entrance and egress corridors for healthcare workers and patients to ensure complete physical and airflow separation, establish multistep, interlocked ventilation zones for healthcare workers to put on the required personal protective equipment, as well as a similarly tiered degowning procedures with final shower-out. Patients were received in a designated buffer area (in some cases containing automatic-spray cleaning systems to sanitize between patients) with dedicated elevators and corridors for patient flow. All negative-pressure isolation rooms were designed with single-pass (100% outside air with no recirculation) dedicated exhaust systems. Exhaust air in all hospitals was treated with HEPA filtration and UVGI before discharge. Visual indicators at the IR door and a remote indicator panel in the nurses' station monitored isolation room pressure. Isolation design included a pressure gradient from the clean (e.g., nurses' station or change room) to the less clean (patient room), including buffer zones to achieve the desired conditions (Figure 3). Designated laundry and medical waste corridors were established, and aggressive cleaning regimens were implemented to ensure frequent sanitation of all areas, including twice daily cleaning of patient rooms and autoclaving of waste before its removal from the facility. Personal protective equipment requirements varied somewhat among hospitals, but typically healthcare workers wore an N95 or N100 respirator, protective suit, double or triple disposable gloves, shoe covers, outer gown, hair cover or hood, and face shield, goggles, or both (Figure 4). To help healthcare workers alleviate heat stress from the encumbering personal protective equipment, work shifts in some hospitals were reduced to 3 or 4 hours. All hospitals had an established infection control department and an infection control plan. A summary of the key design features of the 11 SARS-dedicated hospitals is listed in Table 4.

**Table 4:** Summary of key features of dedicated SARS hospitals^a,b^

Hospital	No. IR/no. PR^c^	Work shift	Shower out^d^	T&B^e^	% compliance^f^	No. SARS patients
DSH-1	102/un	4 h	Y	Un	100	45
DSH-2	92/210	8 h	Y	Un	100	21
DSH-3	126/un	ND	Y	Un	40	0
DSH-4	40/60	4 h	Y	Un	100	0
DSH-5	42/80	3-2-3 h	Y	Y	95	0
DSH-6	47/108	4 h	N	Un	60	0
DSH-7	81/100	4 h	Y	Y	95	0
DSH-8	72/un	3 h	Y	Un	70	0
DSH-9	78/un	3 h	Y	Un	90	0
DSH-10	28/49	3-2-3 h	Y	Un	60	0
DSH-11	32/100	8 h	Y	Un	100	0

^a^All hospitals had a separate healthcare worker and patient path, single-pass air-handling units that provided 100% outside air with no recirculation, HEPA-filtered exhaust systems for the isolation rooms, UV light germicidal irradiation in the exhaust systems, and visible continuous negative pressure monitor with alarm that demonstrated the isolation room is operating under negative pressure. ^b^SARS, severe acute respiratory syndrome; DSH, dedicated SARS hospital; un, unknown or unavailable at the time of the investigation; Y, yes; N, no; ND, not done. ^c^Number of isolation rooms (IR) constructed and number of patient rooms (PR) previously at the hospital. ^d^Personnel exiting the isolation ward are required to shower as part of the change-out protocol. ^e^Facility has completed a test and balance (T&B) (commissioning) of the ventilation system to verify proper function. ^f^Percentage of the construction completed.

Although the major reconfiguration design goals were the same for all hospitals, structural realities and other practical considerations resulted in differences in final configuration. Design changes during construction were also necessary to overcome unforeseen engineering obstacles. Examples of differences include the following: 2 of 11 hospitals did not have elevator capability for healthcare workers to access isolation wards, 5 of 11 hospitals did not have positive-pressure nurses' stations in the isolation ward, not all hospitals had anterooms, and 3 of 11 hospitals did not have complete patient and healthcare worker pathway separation. Other differences included the number of UVGI units in the ventilation system, whether the UVGI was located in front of or behind the HEPA or prefilter, the type (electronic or mechanical) and location of room pressure monitors, the redundancy strategy (dual exhaust, dual filter, or both), and whether the ventilation system received testing and balancing after installation.

## Conclusions

From a facility, personnel, environmental and occupational health perspective, the response to SARS in Taiwan had a profound impact on the healthcare system of the nation. The Taiwanese government responded in a swift and comprehensive manner to contain the outbreak. Although major gaps in knowledge existed regarding this first emerged infectious disease of the 21st century, decisions involving massive resource commitments had to be made quickly and decisively. Hospitals and medical centers islandwide renovated their facilities rapidly or constructed new patient treatment facilities to contain and treat known or suspected SARS patients. Healthcare workers learned to use personal protective equipment in a far more judicious and extensive manner than they were accustomed to. Large-scale retraining and reassignment of thousands of healthcare personnel was also required.

When SARS or a SARS-like pandemic recurs, industrial hygiene specialists will be faced with similar circumstances and should anticipate that they will be forced to respond in a highly charged environment of considerable scientific uncertainty. While the standard industrial hygiene rubrics of anticipation, recognition, evaluation, and control remain useful, more inventive approaches to risk and hazard assessment will be necessary and will test responders' capabilities and tenacity. Some of the challenges these environmental specialists encountered during the SARS response included the following: developing expedient guidelines for engineering and administrative controls for workers and workplaces; developing personal protective equipment use guidelines for healthcare workers and the general public, including questions regarding the feasibility of disinfection and reuse and of disposable respirators; developing and providing training on personal protective equipment use by workers, especially healthcare workers; evaluating hospital isolation rooms and ventilation systems, including containment of window air-conditioner condensate from SARS patient rooms; working with hospital infection control and facilities personnel to develop alternative triage facilities, such as fever screening clinics; advising facilities design personnel on hospital reconfiguration to improve patient transport and patient isolation; developing, applying, and interpreting results from unvalidated and novel environmental sampling techniques; creating effective risk communication tools for workers and the general public; advising local officials on issues of isolation, quarantine, and other public safety concerns, including obtuse inquiries such as the utility of disinfecting septic systems contaminated with SARS-CoV, and evaluating the feasibility of novel patient containment and treatment facilities, and the use of unproven, yet theoretically reasonable control technologies in an emergency situation.
